# Discrimination ability of central visual field testing using stimulus size I, II, and III and relationship between VF findings and macular ganglion cell thickness in chiasmal compression

**DOI:** 10.1371/journal.pone.0300103

**Published:** 2024-03-08

**Authors:** Arthur Andrade do Nascimento Rocha, Thais de Souza Andrade Benassi, Luiz Guilherme Marchesi Mello, Rony Carlos Preti, Leandro C. Zacharias, Leonardo P. Cunha, Mário L. R. Monteiro

**Affiliations:** 1 Division of Ophthalmology and the Laboratory for Investigation in Ophthalmology (LIM-33), Faculdade de Medicina FMUSP, Universidade de São Paulo, São Paulo, Brazil; 2 Division of Ophthalmology, Hospital Universitário Cassiano Antônio Moraes (HUCAM-EBSERH), Universidade Federal do Espírito Santo, Vitória, Brazil; 3 Department of Ophthalmology, Federal University of Juiz de Fora Medical School, Juiz de Fora, Minas Gerais, Brazil; University of Florida College of Medicine, UNITED STATES

## Abstract

**Purpose:**

To compare the relationship between macular ganglion cell layer (mGCL) thickness and 10–2 visual field (VF) sensitivity using different stimulus sizes in patients with temporal hemianopia from chiasmal compression.

**Methods:**

A cross-sectional study was conducted involving 30 eyes from 25 patients with temporal VF loss on 24–2 SITA standard automated perimetry due to previous chiasmal compression and 30 healthy eyes (23 controls). Optical coherence tomography (OCT) of the macular area and 10–2 VF testing using Goldmann stimulus size I (GI), II (GII), and III (GIII) were performed in the Octopus 900 perimeter. For the sake of analysis, mGCL thickness and VF data were segregated into four quadrants (two temporal and two nasal) and two halves (temporal and nasal) centered on the fovea, in order to evaluate separately both the severely affected nasal hemi-retina corresponding to the temporal VF sectors and the subclinically affected temporal hemi-retina corresponding to the nasal VF sectors. Data from patients and controls were compared using generalized estimated equations. The discrimination ability of GI, GII, and GIII was evaluated, as was the correlation between mGCL and 10–2 VF sensitivity using GI, GII, and GIII.

**Results:**

All mGCL parameters in the nasal and temporal halves of the retina were significantly reduced in patients compared to controls. 10–2 VF test sensitivity using GI, GII, and GIII was significantly lower in patients than in controls (p≤0.008) for all parameters, except the three nasal divisions when using GI (p = 0.41, 0.07 and 0.18) Significant correlations were found between temporal VF sectors (all stimulus sizes) and the corresponding nasal mGCL measurements, with similar discrimination ability. Significant correlations were also observed between all three nasal VF divisions and the corresponding temporal mGCL thickness when using stimulus sizes I and II, but not stimulus size III.

**Conclusions:**

On 10–2 VF testing, GII outperformed GI and GIII with regard to discrimination ability and structure-function correlation with mGCL thickness in the subclinically affected nasal part of the VF in patients with chiasmal compression. Our findings suggest that the use of GII can enhance the diagnostic power of 10–2 VF testing in early cases of chiasmal compression, although further studies are necessary to support this conclusion.

## Introduction

Evaluation of structure-function relationships is crucial for the diagnosis and management of anterior visual pathway diseases, such as glaucomatous, inflammatory, degenerative, ischemic, and compressive optic neuropathies [1–5]. Structural assessment typically involves measuring the thickness of the peripapillary retinal nerve fiber layer (pRNFL) and the macular ganglion cell layer (mGCL) [6], while functional measures are usually obtained by standard automated perimetry (SAP) using the 30–2 strategy or the 24–2 strategy, both of which with test points spaced 6° apart, or the central 10–2 strategy, which employs a 2° grid [6].

Advances in optical coherence tomography (OCT) technology have yielded significant improvements in retinal image resolution, providing more accurate mGCL measurements. This has made the central 10–2 SAP strategy the preferred method of evaluating the relationship between OCT-measured macular parameters and visual field (VF) data [6–10], rather than the 6° grid strategies (24–2 and 30–2). However, even with the improvement of the spatial correspondence between mGCL thickness and 10–2 VF data, discordance between structural and functional damage may still occur, leading to difficulties in the diagnosis and management of glaucoma [7, 11] and other optic nerve diseases, including compressive and hereditary optic neuropathies [12, 13].

Most investigations evaluating VF on SAP have used the white-on-white Goldmann size III (GIII) stimulus as standard to measure differential light sensitivity (DLS) [14–16]. DLS, the ratio of background luminance to target luminance at threshold, is used to assess retinal ganglion cell loss as a functional measurement of optic nerve damage. DLS obtained with different stimulus sizes is thought to be related to the size of the receptive field of the retinal ganglion cells [17]. GIII has been used on SAP in numerous clinical and experimental studies [14, 16, 18], though more often out of habit and for historical than for psychophysical reasons [11, 15, 18].

Previous studies have suggested that GIII may exceed the critical summation area of the central VF, reducing its sensitivity to detect subtle defects [11, 19]. Yoshioka et al. [11] demonstrated in glaucoma subjects that smaller stimuli, such as Goldmann sizes I (GI) and II (GII), were more likely to fit the spatial summation in less eccentric areas, and would therefore increase chances of early detection of central VF dysfunctions. Other studies have confirmed that smaller stimulus sizes are more sensitive for the detection of glaucomatous defects [20, 21]. However, to our knowledge, no previous study has evaluated the ability of non-GIII VF stimuli to assess non-glaucomatous neuropathies [11]. Moreover, studies have shown that subjects with chiasmal compression may experience significant mGCL loss despite mild or no central VF impairment [12, 13], suggesting that GIII is not the most sensitive stimulus for the detection of VF defects in patients with subtle mGCL damage.

Subjects with chiasmal compression and band atrophy (BA) of the optic nerve [22] provide a good model for structure-function relationship studies due to the preferential loss of the crossed chiasmal fibers that correspond to significantly affected temporal VF sectors and a relative sparing of the uncrossed fibers, that correspond to mildly affected VF sectors. In addition, the fact that pituitary adenomas are the most common intracranial tumors causing visual loss makes VF testing an important diagnostic and management tool [23]. Since SAP is the most widely used method of VF assessment, enhancing its sensitivity to detect early optic pathway disorders will benefit patient care [7].

The purpose of this study was to evaluate the diagnostic performance of 10–2 VF testing using GI, GII, and GIII stimuli, and to compare the correlation between mGCL thickness and DLS for each stimulus size in both the temporal and the nasal hemifield of patients with temporal hemianopia from previous chiasmal compression. Our purpose was to investigate which of the three stimulus sizes was best able to discriminate between healthy controls and patients with subtle changes in the less-affected part of the VF due to compressive optic neuropathy and to determine the best correlation with mGCL thickness.

## Methods

### Subjects

This observational cross-sectional study included 30 eyes of 25 individuals with temporal VF defects in the 24–2 Swedish Interactive Thresholding Algorithm (SITA) Standard test due to chiasmal compression from previously treated pituitary adenomas (BA group) and 30 eyes of 23 healthy controls (HC group). The study protocol was approved by the Institutional Review Board Ethics Committee at our institution (*CAPPesq*, Approval Number: 04557018.0.0000.0068) and was compliant with the principles of the Declaration of Helsinki. Informed consent was obtained from all participants. The study was sent to the Ethics committee on December 4, 2018, and after its approval, the data was collected from February 1, 2019 to March 30, 2021.

### Inclusion and exclusion criteria

All participants were enrolled in the study after undergoing a complete ophthalmologic examination, including 24–2 SITA Standard VF testing [Humphrey Field Analyzer (HFA), Carl Zeiss Meditec, Dublin, CA]. In both groups the inclusion criteria were: age above 18 years, spherical refractive error within ± 6 diopters (D), cylinder refraction within 3 D, best-corrected visual acuity ≥ 20/30, and reliable VF testing. To be considered reliable, VF tests had to display less than 25% fixation loss, false-positive, and false-negative responses. Eyes with media opacities, retinal or other optic nerve diseases, uveitis, and complicated ocular surgeries were excluded from the study.

Individuals in the BA group had a history of chiasmal compression from pituitary adenoma at the time of diagnosis, documented with magnetic resonance imaging, followed by effective optic pathway decompression, and with a stable VF defect for at least 6 months prior to enrollment. In this group, the 24–2 VF defect was required to be restricted to the temporal hemifield in the pattern deviation plot, with at least two contiguous non-edge test points (one with p < 0.5% and one with p < 2%) and a presumably normal nasal hemifield (defined as the absence of clusters of 3 or more points, with p < 5% on the pattern deviation plot). The HC group consisted of healthy subjects recruited from among hospital staff, with a normal 24–2 VF (i.e., labeled as “within normal limits” by the manufacturer).

### 10–2 visual field testing

**Fig 1:**
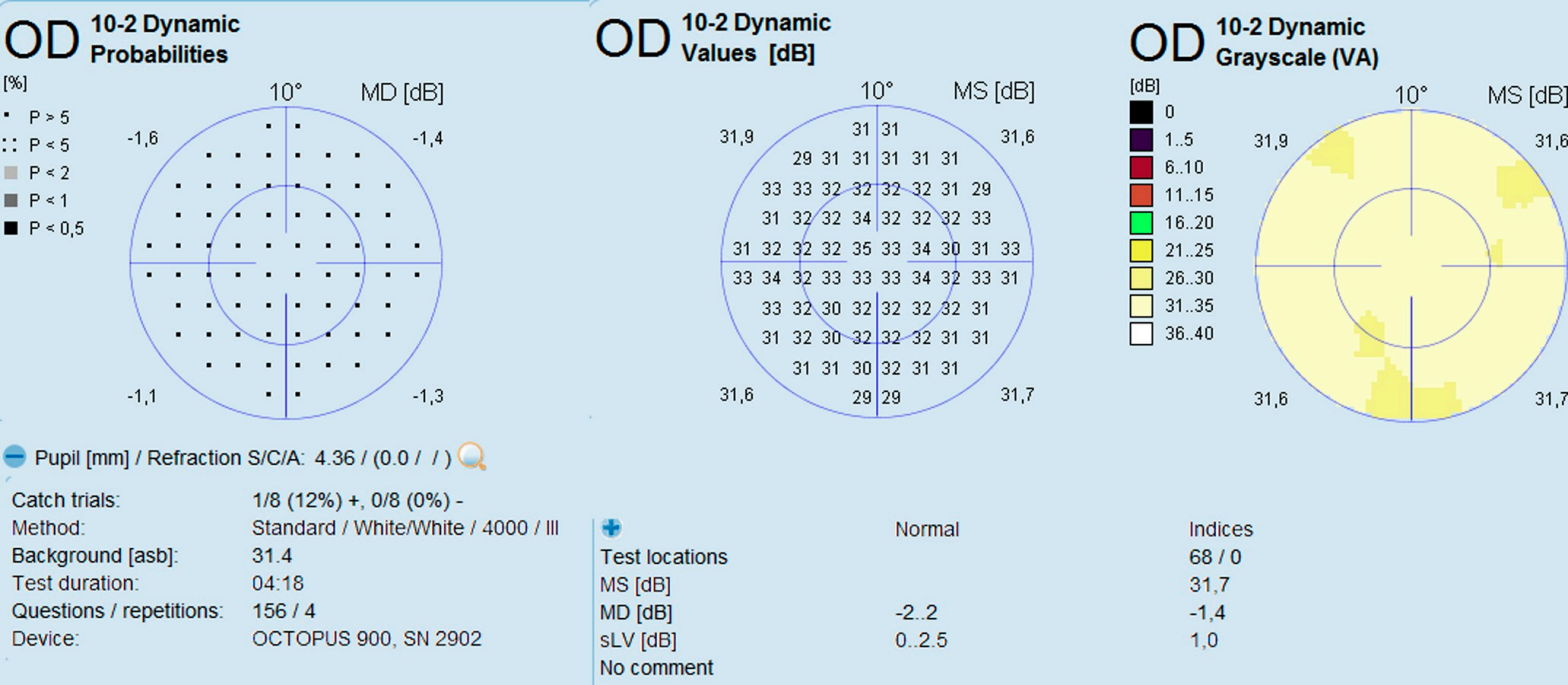
Example of the report of the 10–2 visual field test with size III stimulus performed on a healthy control. Similar visual field tests were obtained with stimulus sizes I and II. Left: graph displaying the probability plot of each test compared to the software database. In this example, all test points were within normal limits (p > 0.05). Center: graph displaying the sensitivity, in decibels, in each of the 68 test points. Right: a colored graphic representation of the sensitivity in the 68 test points.

Under the supervision of the examiner, all participants underwent three sessions of 10–2 VF testing on the Octopus 900 perimeter (Haag Streit International, Koeniz, Switzerland) using the Dynamic strategy, 31.4 apostilbs, white-on-white, and three different Goldmann size stimuli (III, II, and I) (Fig 1). The sessions were separated by 15-min rest breaks.

In the primary analysis, we calculated the average DLS of the 68 test points obtained with each stimulus size (GI, GII, and GIII), averaging the 34 test points in the nasal hemifield (NHF), the 34 test points in the temporal hemifields (THF), and the 17 test points in each of the four quadrants: superotemporal (ST), inferotemporal (IT), superonasal (SN), and inferonasal (IN). DLS values were measured in decibels and converted to 1/Lambert linear units by dividing the dB value by 10 and anti-logging the quotient for average calculations. The mean DLS for each VF parameter was then converted back to decibel units.

### OCT imaging

**Fig 2:**
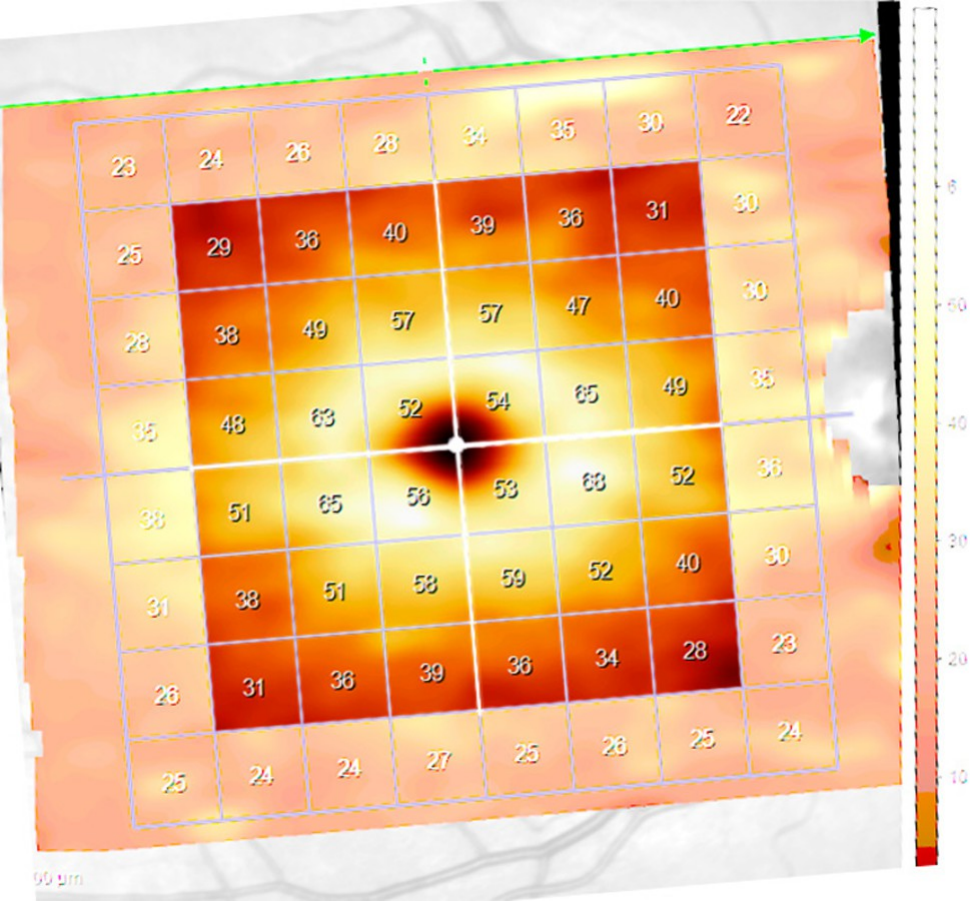
Demarcation of the macular area of a right eye scanned by optical coherence tomography for the measurement of ganglion cell layer thickness following retinal layer segmentation. The color-enhanced squares represent the analyzed area, divided into four quadrants and two hemifields (white lines). The peripheral squares were excluded from the analysis. Note the inclination of the scan due to fovea-disc alignment.

After a comprehensive ophthalmological examination, including VF testing, the subjects’ pupils were dilated with 1% tropicamide for high-resolution macular OCT scanning (Spectralis^®^ OCT-2, Heidelberg Engineering, Heidelberg, Germany) using the Horizontal Posterior Pole protocol available in the Glaucoma Module Premium Edition. The images were acquired using automated eye alignment eye-tracking software (TruTrack; Heidelberg Engineering) and corrected with the Fovea-to-Disc Alignment system to obtain macular volumetric retinal scans comprising 61 single vertical lines of 16 frames each, with a scan rate of 85,000 Hz, covering a cuboid area of 30° x 25° (9.2 x 7.6 mm) centered on the fovea. A central 6 x 6 mm square of the scanned area was used for analysis (Fig 2). The software scores the quality of the signal strength of the images on a scale from poor (0 dB) to excellent (40 dB). A minimum quality index of 20 was required for all images. All images were reviewed for subjective and objective quality.

The seven retinal layers were automatically segmented by the Spectralis software and manually corrected as needed: the macular retinal nerve fiber layer, the ganglion cell layer (GCL), the inner plexiform layer, the inner nuclear layer, the outer plexiform layer, the outer nuclear layer, and the photoreceptor layer [24]. For the present study, we registered the average GCL thickness of the 36 central squares of the macular area segmented into hemifields (NHR, THR) and quadrants (IN, IT, SN, ST). The peripheral squares were excluded from the analysis (Fig 2).

### Statistics

**Fig 3:**
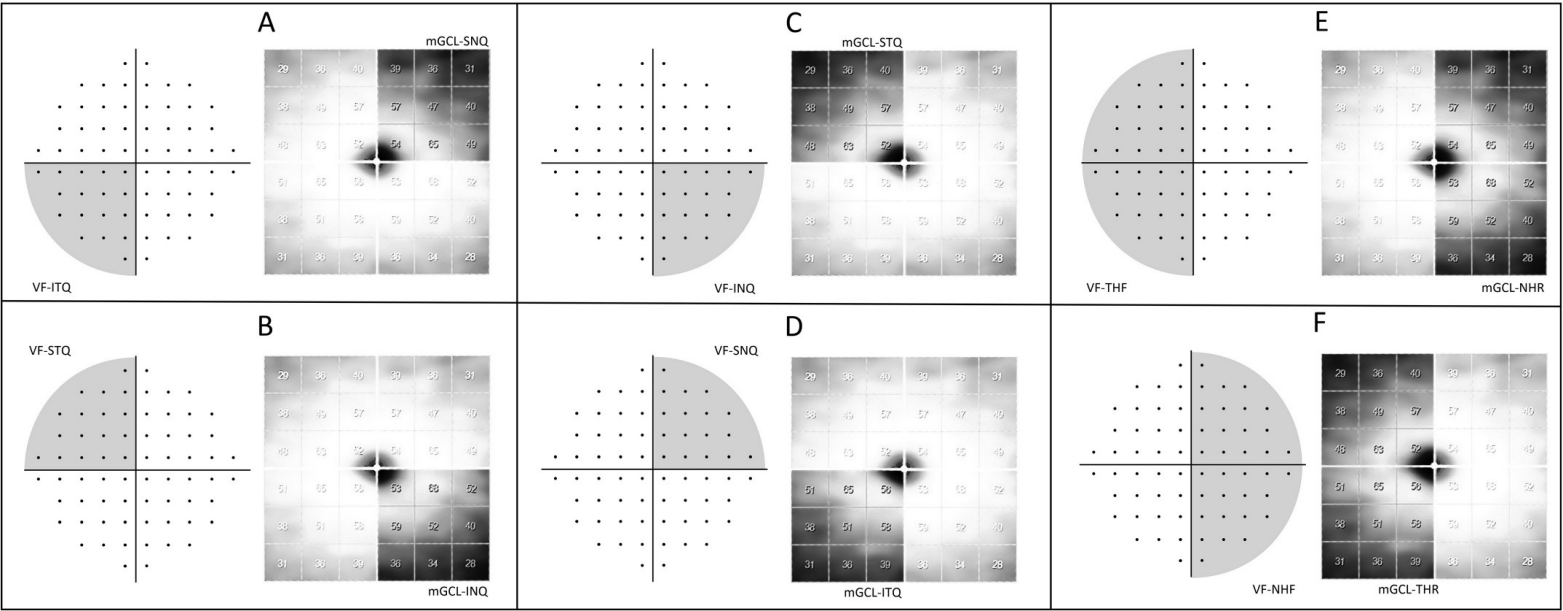
Optical coherence tomography sectors (right column) of the macular ganglion cell layer (mGCL) of a right eye and the corresponding 10–2 visual field (VF) area (left column). The areas of interest are represented in dark gray. The inferotemporal and superotemporal visual field quadrants (ITQ and STQ, respectively) were correlated with the superonasal (A) and inferonasal (B) mGCL sectors, respectively, while the inferonasal and superonasal visual field quadrants (INQ and SNQ, respectively) were correlated with the superotemporal (C) and inferotemporal (D) mGCL sectors, respectively. The temporal and nasal hemifields (THF and NHF) were correlated, respectively, with the nasal and temporal hemiretinas (NHR and THR, respectively, E and F).

The statistical analysis was performed using the software IBM SPSS Statistics v. 25. The distribution of the data was verified with the Kolmogorov-Smirnov test for normality. Descriptive parameters were expressed as mean values ± standard deviation for normally distributed variables. The two groups (BA and HC) were compared with regard to mGCL thickness and 10–2 VF parameters using generalized estimated equations (GEE) to account for inter-eye dependency. Receiver operating characteristic (ROC) curves were used to assess the discrimination ability of each parameter. Comparisons between areas under ROC curves (AUCs) were conducted using the method of DeLong et al. [25]. Correlations between OCT data and the corresponding DLS sector (in 1/L units) of the 10–2 VF using stimuli size I, II and III (Fig 3) were assessed using Spearman’s ranked correlation coefficients (*ρ*). The level of statistical significance was set at 5% *(*p < 0.05).

## Results

A total of 30 eyes from 25 individuals with band atrophy of the optic nerve from chiasmal compression and 30 eyes from 23 healthy controls were evaluated. There was no statistically significant difference between the groups with regard to the collected demographic data, but all 24–2 VF parameters were significantly reduced in the BA group (Table 1). VF analysis shows that patients presented not only severely reduced temporal VF sensitivity but also mildly reduced nasal VF sensitivity (Table 1). The mGCL (all sectors) was also thinner in the BA group than in the HC group (Table 2). mGCL loss showed greater severity in the nasal hemi-retina and quadrants (approximately 15 microns), while it was only mildly to moderately reduced in the temporal hemi-retina and quadrants (approximately 7 microns).

**Table 1:** Demographic data and the 24–2 visual field deviations analysis (24–2 VFD) of patients with band atrophy of the optic nerve from chiasmal compression (BA) and healthy controls (HC).

	BA	HC	p-value
**Subjects, (eyes)**	25 (30)	23 (30)	-
**Age, years (mean** ± **SD)**	55.6 ± 16.4	46.4 ± 10.6	0.07*
**Gender (female/male)**	10/15	10/13	0.81**
**Laterality, n (%): OD, OS, OU**	12 (50.00), 6 (25.00), 6 (25.00)	15 (62.50), 3 (12.50), 6 (25.00)	0.62***
**24–2 VFD (mean** ± **SD), in decibels**			
**Global mean deviation**	-4.93 ± 2.40	-0.15 ± 1.27	<0.001****
**Nasal hemifield**	-2.88 ± 2.10	-0.29 ± 1.32	<0.001****
**Superonasal quadrant**	-3.27 ± 2.33	-0.34 ± 1.51	<0.001****
**Inferonasal quadrant**	-2.61 ± 2.11	-0.28 ± 1.27	<0.001****
**Temporal hemifield**	-13.85 ± 10.71	-0.04 ± 1.24	<0.001****
**Superotemporal quadrant**	-15.22 ± 9.84	-0.12 ± 1.54	<0.001****
**Inferotemporal quadrant**	-13.99 ± 12.43	0.01 ± 1.05	<0.001****

OD: right eye; OS: left eye; OU: both eyes; SD: standard deviation. *Mann-Whitney test; **chi-square test; *** Fisher’s test; **** Generalized estimated equations (GEE). Significant values (p<0.05) are in bold.

**Table 2:** Macular ganglion cell layer (mGCL) thickness (in micra) in eyes with band atrophy of the optic nerve from chiasmal compression (BA) and healthy controls (HC).

mGCL	BA (mean ± SD)	HC (mean ± SD)	p-value*	AUC (SE)	S/E≥95%	S/E≥80%
Global average	28.56 ± 4.35	39.54 ± 3.60	**<0.001**	0.973 (0.02)	87/97	97/80
Nasal hemiretina	25.42 ± 4.28	40.28 ± 3.80	**<0.001**	0.993 (0.01)	97/97	100/80
Temporal hemiretina	31.70 ± 5.40	38.80 ± 3.54	**<0.001**	0.861 (0.05)	57/97	77/80
ST quadrant	30.93 ± 5.73	38.57 ± 3.87	**<0.001**	0.849 (0.05)	53/97	70/80
IT quadrant	32.48 ± 5.41	39.03 ± 3.33	**<0.001**	0.838 (0.05)	57/97	77/80
SN quadrant	25.74 ± 4.83	40.44 ± 4.02	**<0.001**	0.982 (0.02)	90/97	97/80
IN quadrant	25.09 ± 3.96	40.12 ± 3.67	**<0.001**	0.996 (0.01)	97/97	100/80

AUC: area under the receiver operating curve; IN: inferonasal; IT: inferotemporal; MS: mean sensitivity; NH: nasal hemifield; S/E ≥ 80%: sensitivity when specificity ≥ 80%; S/E ≥ 95%: sensitivity when specificity ≥ 95%; SD: standard deviation; SE: sensitivity; SN: superonasal; ST: superotemporal.

*Generalized estimated equations (GEE). Significant values (p<0.05) are in bold.

Table 3 compares quadrantic and hemifield DLS in BA and CT obtained with the 10–2 VF strategy using size I, II, and III stimuli. Statistically significant differences in DLS were found between BA and HC for all parameters, except SN, IN and NHF using size I stimulus (p = 0.41, 0.07, and 0.18, respectively). No statistically significant difference in AUC was observed when comparing the same sector exposed to stimuli of different sizes.

**Table 3:** Comparison of differential light sensitivity (DLS, in decibels) on 10–2 visual field testing using Goldmann size III, II, and I stimuli in eyes with band atrophy of the optic nerve from chiasmal compression (BA) and healthy controls (HC).

Parameter	BA	HC	p-value*	AUC (SE)	S/E≥95%	S/E≥80%
	DLS (mean ± SD)					
**Size III stimulus**						
ST quadrant	22.65 ± 5.21	29.71 ± 1.62	**<0.001**	0.96 (0.03)	80/97	97/80
IT quadrant	20.23 ± 8.92	29.93 ± 1.52	**<0.001**	0.94 (0.03)	74/97	87/80
Temporal hemifield	21.44 ± 6.48	29.82 ± 1.53	**<0.001**	0.96 (0.02)	84/97	97/80
SN quadrant	28.19 ± 1.81	29.69 ± 1.80	**0.001**	0.74 (0.07)	27/97	63/80
IN quadrant	27.83 ± 1.89	29.89 ± 1.44	**<0.001**	0.80 (0.06)	40/97	70/80
Nasal hemifield	28.01 ± 1.80	29.79 ± 1.58	**<0.001**	0.78 (0.06)	37/97	70/80
**Size II stimulus**						
ST quadrant	19.05 ± 6.32	27.21 ± 1.64	**<0.001**	0.95 (0.02)	74/97	90/80
IT quadrant	16.81 ± 9.49	27.26 ± 1.53	**<0.001**	0.91 (0.04)	77/97	87/80
Temporal hemifield	17.93 ± 7.57	27.24 ± 1.51	**<0.001**	0.94 (0.03)	77/97	87/80
SN quadrant	25.24 ± 2.86	26.97 ± 1.94	**0.008**	0.68 (0.07)	20/97	43/80
IN quadrant	24.73 ± 3.09	27.13 ± 1.60	**<0.001**	0.73 (0.07)	33/97	67/80
Nasal hemifield	24.98 ± 2.88	27.05 ± 1.71	**0.001**	0.72 (0.07)	33/97	60/80
**Size I stimulus**						
ST quadrant	13.42 ± 7.18	21.58 ± 2.39	**<0.001**	0.89 (0.05)	63/97	83/80
IT quadrant	11.71 ± 7.90	21.03 ± 4.34	**<0.001**	0.90 (0.05)	57/97	90/80
Temporal hemifield	12.56 ± 7.18	21.30 ± 2.27	**<0.001**	0.90 (0.04)	53/97	93/80
SN quadrant	19.92 ± 2.82	20.83 ± 4.36	0.41	0.66 (0.07)	30/97	53/80
IN quadrant	19.26 ± 3.26	21.23 ± 4.41	0.07	0.73 (0.07)	37/97	57/80
Nasal hemifield	19.59 ± 2.80	21.03 ± 4.34	0.18	0.71 (0.07)	30/97	53/80

AUC: area under the receiver operating curve; IN: inferonasal; IT: inferotemporal; NH: nasal hemifield; S/E ≥ 80%: sensitivity when specificity ≥ 80%; S/E ≥ 95%: sensitivity when specificity ≥ 95%; SD: standard deviation; SE: sensitivity; SN: superonasal; ST: superotemporal; TH: temporal hemifield.

*Generalized estimated equations (GEE). Significant values (p<0.05) are in bold.

**Fig 4:**
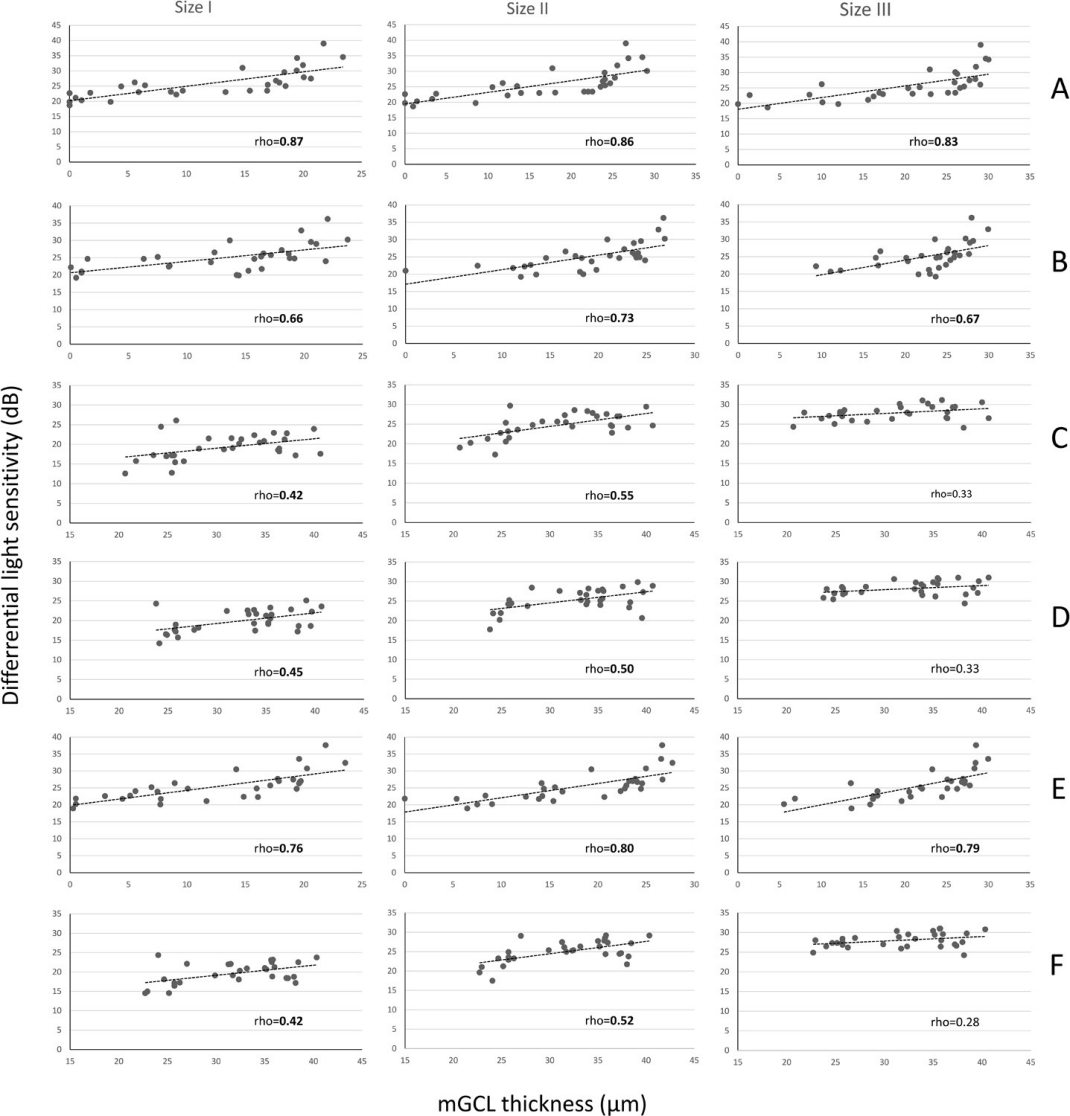
Scatterplots of the correlation between the differential light sensitivity (in decibels) in visual field (VF) testing with Goldmann stimulus size I, II, and III and macular ganglion cell layer (mGCL) thickness in the corresponding sector in eyes with chiasmal compression. A: Inferotemporal VF quadrant *versus* superonasal mGCL quadrant; B: Superotemporal VF quadrant *versus* inferonasal mGCL quadrant; C: inferonasal VF quadrant *versus* superotemporal mGCL quadrant; D: superonasal VF quadrant *versus* inferotemporal mGCL quadrant; E: temporal hemifield *versus* nasal hemiretina; F: nasal hemifield *versus* temporal hemiretina. Spearman correlation coefficients (rho) are indicated in each graphic and statistically significant values (p < 0.05) are displayed in bold type.

Correlations between mGCL thickness and the corresponding sectoral DLS for each stimulus size (GI, GII, and GIII) are shown in Table 4. All three stimulus sizes yielded a statistically significant positive correlation between the temporal DLS sector and the nasal mGCL sector. As for the relationship between the nasal DLS sector and the temporal mGCL sector, a statistically significant positive correlation was found for stimuli size GI and GII but not GIII. Fig 4 shows the scatterplots of the correlations between the sectoral DLS using GI, GII, and GIII and mGCL thickness in the corresponding sector in the BA group.

**Table 4:** Correlation between macular ganglion cell layer (mGCL) thickness and the corresponding differential light sensitivity (DLS, in 1/Lambert units) sector of the 10–2 visual field, using Goldmann size III, II, and I stimuli in eyes with band atrophy of the optic nerve from chiasmal compression.

mGCL and the corresponding DLS sector	Size III stimulus	Size II stimulus	Size I stimulus
	rho* (p-*value*)	*rho* (p-*value*)	*rho* (p-*value*)
mGCL-SNQ x DLS-ITQ	**0.83 (<0.001)**	**0.86 (<0.001)**	**0.87 (<0.001)**
mGCL-INQ x DLS-STQ	**0.67 (<0.001)**	**0.73 (<0.001)**	**0.66 (<0.001)**
mGCL-STQ x DLS-INQ	0.33 (0.073)	**0.55 (0.002)**	**0.42 (0.021)**
mGCL-ITQ x DLS-SNQ	0.33 (0.071)	**0.50 (0.005)**	**0.45 (0.012)**
mGCL-NHR x DLS-THF	**0.79 (<0.001)**	**0.80 (<0.001)**	**0.76 (<0.001)**
mGCL-THR x DLS-NHF	0.28 (0.128)	**0.52 (0.003)**	**0.42 (0.021)**

INQ: inferonasal quadrant; ITQ: inferotemporal quadrant; NHF: nasal hemifield; NHR: nasal hemiretina; SNQ: superonasal quadrant; STQ: superotemporal quadrant; THF: temporal hemifield; THR: temporal hemiretina.

*Spearman’s rank correlation coefficient. Statistically significant values (p<0.05) are in bold.

## Discussion

Several authors have argued that SAP using the standard GIII stimulus is relatively insensitive at detecting early optic pathway damage from glaucoma and compressive optic neuropathies based on the observation that structural abnormalities (especially mGCL loss) can precede the development of VF defects [11, 13, 19, 26]. With the availability of modern OCT equipment capable of providing detailed measurements of all retinal layers in the macula, a number of studies have found mGCL abnormalities on OCT despite the absence of abnormalities in VF tests using the standard GIII stimulus [12, 13, 26].

Several strategies and stimuli are employed in VF testing, but the demonstration of mGCL thinning as an early finding in several optic neuropathies has led to the adoption of the 10–2 threshold test to better define the DLS in the corresponding area of the mGCL. When evaluating the structure-function relationship between VF and mGCL, the central 10–2 threshold test provides a significantly better correlations between these parameters, while the 24–2 or 30–2 tests have been shown to miss central VF defects [6, 27]. However, little attention has been given to assessing stimulus sizes other than the standard GIII stimulus.

In our study, we compared the diagnostic efficacy of 10–2 VF testing using GI, GII, and GIII. To do so, we selected patients previously treated for chiasmal compression with a temporal hemifield defect on 24–2 VF, a nasal 24–2 hemifield qualifying as “within normal limits”, and different amounts of mGCL thinning. Matching the findings of previous studies [10, 28], we found that the 24–2 VF sensitivity of both the temporal and the nasal hemifield was significantly lower in BA than in HC. In other words, the nasal hemifield of our patients was classified as being “within normal limits” on 24–2 SAP based on clinical criteria, but the mean sensitivity was significantly lower in BA than in CT, indicating subclinical loss. Using high-resolution OCT we also confirmed that in the eyes of patients with chiasmal compression, not only the nasal but also the temporal hemiretina had thinner mGCL compared to healthy subjects, a finding already documented in previous studies [10, 29, 30]. Thus, despite the predominant damage to the crossed nasal fibers, the uncrossed temporal fibers also experience some damage when the chiasm is compressed. This pattern of neural loss was well-suited for the purpose of our study as it allowed to evaluate the diagnostic performance of 10–2 VF testing by analyzing sectors with different degrees of VF loss, such as mild (nasal quadrants), moderate (inferior temporal quadrant) and severe (upper temporal quadrant).

Our study revealed significant differences between patients previously treated for chiasmal compression and healthy controls regarding the DLS of the temporal quadrants and temporal hemifield using 10–2 VF testing with all three stimulus sizes (GI, GII, and GIII). These findings were expected and consistent with our patient selection criteria, which required the presence of a temporal VF defect on the 24–2 threshold test. However, the outcome differed when evaluating the nasal VF parameters: 10–2 VF testing effectively differentiated the groups when using GII and GIII, but not when using GI (Table 3). The slight reduction of DLS in the nasal field in the BA group did not reach statistical significance, possibly due to an increase in the standard deviation of the HC group, as reported by other authors [31, 32]. Our findings suggest that GI is inadequate for this analysis as it leads to greater response variability and consequently diminishes the ability of the test to differentiate healthy controls from patients previously treated for chiasmal compression. In contrast, GII and GIII were both efficient at distinguishing the groups, suggesting they are suitable for clinical evaluations of the central 10–2 VF.

To further determine which stimulus size best reflects the extent of structural macular damage in chiasmal compression, we evaluated the correlation between sectoral 10–2 VF DLS with different stimulus sizes and the mGCL thickness of the corresponding sector. In the BA group, mGCL thickness displayed a spectrum of structural damage, ranging from subtle in the temporal hemiretina to severe in the inferonasal quadrant of the macula (Table 2). We found a statistically significant correlation between structure and function for all three stimulus sizes when analyzing mGCL in the nasal hemiretina (upper and lower quadrants and their average) and the temporal VF sectors (upper and lower quadrants and their average). On the other hand, when evaluating the correlation between the less affected nasal VF and the temporal macular parameters we observed a significant correlation between temporal mGCL thickness and nasal VF sectors using GI and GII, but not GIII. The correlation coefficients were also greater for GII than for GI when correlating mGCL thickness in ST with DLS in IN.

Our findings are in agreement with those of Yoshioka et al. [11] who evaluated the relationship between DLS on 10–2 VF testing with Goldmann stimulus size I, II, III, IV, and V and the estimated macular ganglion cell count in glaucomatous and normal eyes. The authors found that the smaller sizes were significantly better correlated with ganglion cell count. DLS reduction was more accurately predicted with GI than with the other sizes, but GI also increased data variability. On the other hand, GII predicted a significantly smaller ganglion cell count than did GIII and GV without increased variability. Other studies have suggested that smaller stimuli are more sensitive to detecting VF defects since they are smaller than the critical summation area of the retina and thus allow for earlier diagnosis [20, 21]. Among other factors, sensitivity in the detection of VF abnormalities depends on the extent to which retinal eccentricity impacts the correspondence between the VF and the receptive fields of the ganglion cells [14, 16]. The structure-function relationship is determined by differences between the characteristics of spatial summation of the central and peripheral retina. Our findings that GII was better than GI in differentiating patients from controls (Table 3) and that it better correlates with mGCL thickness when compared to GIII (Table 4), strongly suggest that GII (0.22° diameter) provides a better match for the critical summation area of the retinal ganglion cells in the macular area than does GIII (0.43° diameter) [11].

Among the study limitations one may cite the relatively small number of eyes studied and the inclusion of some eyes with severe temporal VF defect on the 24–2 test. On the other hand, the evaluation of differently affected VF quadrants widened the range of disease severity, making it possible to adequately evaluate the relationship with mGCL parameters, thus to some extent compensating for the inclusion of patients with severe temporal VF defects.

In conclusion, the central 10–2 VF testing using stimulus sizes GII and GIII provided a better discrimination ability between patients with chiasm compression and healthy controls than GI. However, GII allowed for better correlations between VF data and the corresponding mGCL thickness than did GIII when evaluating the relationship between the less affected nasal VF and temporal macular parameters. Furthermore, in addition to the classical temporal hemianopic field defect and its corresponding retinal structural damage, the nasal VF sectors and temporal macular ganglion cell layer thickness can also be affected by chiasm compressive lesion, even though the VF tests with size III stimulus are described as “within normal limits” by the equipment database. Overall, the diagnostic performance of 10–2 VF testing was better with GII than with GI, and even better than the widely used GIII stimulus size.

## References

[1] Hood DC, Kardon RH. A framework for comparing structural and functional measures of glaucomatous damage. Prog Retin Eye Res. 2007;26(6):688–710. doi: 10.1016/j.preteyeres.2007.08.001 17889587 PMC2110881

[2] Hood DC, Anderson S, Rouleau J, Wenick AS, Grover LK, Behrens MM, et al. Retinal nerve fiber structure versus visual field function in patients with ischemic optic neuropathy. A test of a linear model. Ophthalmology. 2008;115(5):904–10. doi: 10.1016/j.ophtha.2007.06.001 17870170 PMC2987576

[3] Monteiro ML, Hokazono K, Fernandes DB, Costa-Cunha LV, Sousa RM, Raza AS, et al. Evaluation of inner retinal layers in eyes with temporal hemianopic visual loss from chiasmal compression using optical coherence tomography. Invest Ophthalmol Vis Sci. 2014;55(5):3328–36. doi: 10.1167/iovs.14-14118 24764062 PMC4322661

[4] Danesh-Meyer HV, Carroll SC, Foroozan R, Savino PJ, Fan J, Jiang Y, et al. Relationship between retinal nerve fiber layer and visual field sensitivity as measured by optical coherence tomography in chiasmal compression. Invest Ophthalmol Vis Sci. 2006;47(11):4827–35. doi: 10.1167/iovs.06-0327 17065494

[5] Sousa RM, Oyamada MK, Cunha LP, Monteiro MLR. Multifocal Visual Evoked Potential in Eyes With Temporal Hemianopia From Chiasmal Compression: Correlation With Standard Automated Perimetry and OCT Findings. Invest Ophthalmol Vis Sci. 2017;58(11):4436–49. doi: 10.1167/iovs.17-21529 28863215

[6] Hood DC, Raza AS, de Moraes CG, Liebmann JM, Ritch R. Glaucomatous damage of the macula. Prog Retin Eye Res. 2013;32:1–21. doi: 10.1016/j.preteyeres.2012.08.003 22995953 PMC3529818

[7] Malik R, Swanson WH, Garway-Heath DF. ’Structure-function relationship’ in glaucoma: past thinking and current concepts. Clin Exp Ophthalmol. 2012;40(4):369–80. doi: 10.1111/j.1442-9071.2012.02770.x 22339936 PMC3693944

[8] Raza AS, Cho J, de Moraes CG, Wang M, Zhang X, Kardon RH, et al. Retinal ganglion cell layer thickness and local visual field sensitivity in glaucoma. Arch Ophthalmol. 2011;129(12):1529–36. doi: 10.1001/archophthalmol.2011.352 22159673 PMC4331118

[9] Hood DC, Raza AS, de Moraes CG, Odel JG, Greenstein VC, Liebmann JM, et al. Initial arcuate defects within the central 10 degrees in glaucoma. Invest Ophthalmol Vis Sci. 2011;52(2):940–6. doi: 10.1167/iovs.10-5803 20881293 PMC3053114

[10] de Araujo RB, Oyamada MK, Zacharias LC, Cunha LP, Preti RC, Monteiro MLR. Morphological and Functional Inner and Outer Retinal Layer Abnormalities in Eyes with Permanent Temporal Hemianopia from Chiasmal Compression. Front Neurol. 2017;8:619. doi: 10.3389/fneur.2017.00619 29255441 PMC5723053

[11] Yoshioka N, Zangerl B, Phu J, Choi AYJ, Khuu SK, Masselos K, et al. Consistency of Structure-Function Correlation Between Spatially Scaled Visual Field Stimuli and In Vivo OCT Ganglion Cell Counts. Invest Ophthalmol Vis Sci. 2018;59(5):1693–703. doi: 10.1167/iovs.17-23683 29610852

[12] Monteiro MLR. Macular Ganglion Cell Complex Reduction Preceding Visual Field Loss in a Patient With Chiasmal Compression With a 21-Month Follow-Up. J Neuroophthalmol. 2018;38(1):124–7. 10.1097/WNO.0000000000000625 29319560

[13] Tieger MG, Hedges TR 3rd, Ho J, Erlich-Malona NK, Vuong LN, Athappilly GK, et al. Ganglion Cell Complex Loss in Chiasmal Compression by Brain Tumors. J Neuroophthalmol. 2017;37(1):7–12. doi: 10.1097/WNO.0000000000000424 28192385 PMC6033516

[14] Garway-Heath DF, Caprioli J, Fitzke FW, Hitchings RA. Scaling the hill of vision: the physiological relationship between light sensitivity and ganglion cell numbers. Invest Ophthalmol Vis Sci. 2000;41(7):1774–82. 10845598

[15] Swanson WH. Stimulus size for perimetry in patients with glaucoma. Invest Ophthalmol Vis Sci. 2013;54(6). doi: 10.1167/iovs.13-12335 23743001 PMC4597430

[16] Swanson WH, Felius J, Pan F. Perimetric defects and ganglion cell damage: interpreting linear relations using a two-stage neural model. Invest Ophthalmol Vis Sci. 2004;45(2):466–72. doi: 10.1167/iovs.03-0374 14744886

[17] Glezer VD. The receptive fields of the retina. Vision Res. 1965;5(9):497–525. doi: 10.1016/0042-6989(65)90084-2 5862172

[18] Anderson RS. The psychophysics of glaucoma: improving the structure/function relationship. Prog Retin Eye Res. 2006;25(1):79–97. doi: 10.1016/j.preteyeres.2005.06.001 16081311

[19] Phu J, Khuu SK, Yapp M, Assaad N, Hennessy MP, Kalloniatis M. The value of visual field testing in the era of advanced imaging: clinical and psychophysical perspectives. Clin Exp Optom. 2017;100(4):313–32. doi: 10.1111/cxo.12551 28640951 PMC5519947

[20] Zalta AH, Burchfield JC. Detecting early glaucomatous field defects with the size I stimulus and Statpac. Br J Ophthalmol. 1990;74(5):289–93. doi: 10.1136/bjo.74.5.289 2354137 PMC1042101

[21] Rao HL, Qasim M, Hussain RS, Januwada M, Pillutla LN, Begum VU, et al. Structure-Function Relationship in Glaucoma Using Ganglion Cell-Inner Plexiform Layer Thickness Measurements. Invest Ophthalmol Vis Sci. 2015;56(6):3883–8. doi: 10.1167/iovs.15-16943 26070060

[22] Unsold R, Hoyt WF. Band atrophy of the optic nerve. The histology of temporal hemianopsia. Arch Ophthalmol. 1980;98(9):1637–8. doi: 10.1001/archopht.1980.01020040489020 7425927

[23] Monteiro ML, Zambon BK, Cunha LP. Predictive factors for the development of visual loss in patients with pituitary macroadenomas and for visual recovery after optic pathway decompression. Can J Ophthalmol. 2010;45(4):404–8. doi: 10.3129/i09-276 20648089

[24] Cruz-Herranz A, Balk LJ, Oberwahrenbrock T, Saidha S, Martinez-Lapiscina EH, Lagreze WA, et al. The APOSTEL recommendations for reporting quantitative optical coherence tomography studies. Neurology. 2016;86(24):2303–9. doi: 10.1212/WNL.0000000000002774 27225223 PMC4909557

[25] DeLong ER, DeLong DM, Clarke-Pearson DL. Comparing the areas under two or more correlated receiver operating characteristic curves: a nonparametric approach. Biometrics. 1988;44(3):837–45. 3203132

[26] Horton JC. Invited Commentary: Ganglion Cell Complex Measurement in Compressive Optic Neuropathy. J Neuroophthalmol. 2017;37(1):13–5. doi: 10.1097/WNO.0000000000000489 28187079 PMC5689083

[27] Grillo LM, Wang DL, Ramachandran R, Ehrlich AC, De Moraes CG, Ritch R, et al. The 24–2 Visual Field Test Misses Central Macular Damage Confirmed by the 10–2 Visual Field Test and Optical Coherence Tomography. Transl Vis Sci Technol. 2016;5(2):15. doi: 10.1167/tvst.5.2.15 27134774 PMC4849532

[28] Monteiro ML, Cunha LP, Costa-Cunha LV, Maia OO Jr., Oyamada MK. Relationship between optical coherence tomography, pattern electroretinogram and automated perimetry in eyes with temporal hemianopia from chiasmal compression. Invest Ophthalmol Vis Sci. 2009;50(8):3535–41. doi: 10.1167/iovs.08-3093 19264884

[29] Moura FC, Medeiros FA, Monteiro ML. Evaluation of macular thickness measurements for detection of band atrophy of the optic nerve using optical coherence tomography. Ophthalmology. 2007;114(1):175–81. doi: 10.1016/j.ophtha.2006.06.045 17070583

[30] Monteiro ML, Costa-Cunha LV, Cunha LP, Malta RF. Correlation between macular and retinal nerve fibre layer Fourier-domain OCT measurements and visual field loss in chiasmal compression. Eye (Lond). 2010;24(8):1382–90. doi: 10.1038/eye.2010.48 20431609

[31] Spry PG, Johnson CA, McKendrick AM, Turpin A. Variability components of standard automated perimetry and frequency-doubling technology perimetry. Invest Ophthalmol Vis Sci. 2001;42(6):1404–10. 11328758

[32] Wall M, Kutzko KE, Chauhan BC. Variability in patients with glaucomatous visual field damage is reduced using size V stimuli. Invest Ophthalmol Vis Sci. 1997;38(2):426–35. 9040476

